# Evaluating Digital Photography for Lip Print Recording: Compatibility With Traditional Classification Systems

**DOI:** 10.7759/cureus.58238

**Published:** 2024-04-14

**Authors:** Renjith George Pallivathukal, Sameer Kumar, Jose Joy Idiculla, Htoo Htoo Kyaw Soe, Yen Ying Ke, Preethy Mary Donald, Noorliza Mastura Ismail

**Affiliations:** 1 Oral Pathology, Manipal University College Malaysia, Melaka, MYS; 2 Community Medicine, Manipal University College Malaysia, Melaka, MYS; 3 Oral Medicine and Oral Radiology, Manipal University College Malaysia, Melaka, MYS; 4 Community Dentistry, Manipal University College Malaysia, Melaka, MYS

**Keywords:** forensic dentistry, digital images, lip print pattern, cheiloscopy, lip print

## Abstract

Background: Direct digital photography for lip print recording is a recent trend, and there is a dearth of systematic research on the analysis of the recorded prints with existing clip print classification systems.

Aims and objectives: This study aims to compare the accuracy of the digital photographic method in lip print recording, comparing it with traditional methods, and assessing the suitability of commonly used lip print classification for analyzing lip prints recorded by photographic method.

Methodology: A total of 72 participants aged between 20 and 26 were included. The lip print recording process involved photographing the lips without and with lipstick, followed by recording the lip print with cellophane tape on bond paper. The prints collected using the different methods were analyzed and compared for agreement, and the data were analyzed statistically.

Results: Inter-observer reliability was high for all methods (>0.800). The distribution of lip print patterns differed across the methods, suggesting a potential influence of the recording technique. The agreement between the conventional method and both digital methods was moderate (kappa=0.449-0.517). The agreement between digital methods with and without enhancement was also moderate (kappa=0.718). Notably, digital photographs with enhancement tend to have a higher positive agreement for several lip print types.

Conclusion: Digital photography is a potential method for lip print recording. However, this study highlights the need for the calibration of lip print classification systems for digitally recorded lip prints. Further research is needed to refine the use of digital photography in forensic lip print analysis and to explore its integration with artificial intelligence for biometric identification.

## Introduction

Lip print analysis, an important aspect of cheiloscopy in forensic science, has been a subject of extensive research since its initial identification and classification. The scientific literature on cheiloscopy encompasses numerous publications on the classification, prevalence, sexual dimorphism, and ancestral patterns of lip prints [1]. However, research focusing on the methods employed for lip print recording in a research context remains relatively underexplored. Several methods have been documented in the literature for lip print recording, including photography, photographic enlargement, and overlay tracings on non-porous surfaces such as mirrors, application of transfer media such as lipstick or lip rouge followed by lip impression on paper or tape, utilization of finger printers (preferably roller finger printers), and lip impression on suitable surfaces processed with conventional fingerprint development powder or magna brush and magnetic powder [2]. A review of the literature reveals that the conventional method of lip print recording, involving the use of lip print enhancers (e.g., lipstick, lip gloss), lifting prints with adhesive tape (such as cellophane or clear tape), and pasting them onto contrasting backgrounds (typically white paper), followed by manual or digital analysis, is the predominant approach [3]. Digital photography offers a modern alternative to lip print recording. Unlike traditional methods, digital photography allows high-resolution image capture, thus enabling the preservation of finer lip patterns and details. However, despite its potential advantages, there is a gap in research regarding the systematic use of digital photography in lip print recording and analysis. 

Several studies have compared the accuracy of digital photography with traditional methods for recording lip prints, with results suggesting that digital photography is as accurate, if not more accurate than traditional methods. A previous study found that digital photography was more accurate than lipstick and cellophane tape methods for recording lip prints and was more accurate for females than males [3]. Another study by Prabhu et al. found that digital photography was as accurate as the inkless method for recording lip prints and was more efficient and less time-consuming than the inkless method [4]. Additionally, Hamzah et al. compared the accuracy of digital photography with and without the application of lipstick and found that digital photography was more accurate with lipstick application and that the type of lipstick used affected the accuracy of digital photography [5].

The use of cellophane and lipsticks to record lip prints, followed by scanning and digitalization, is a common method in forensic sciences and biometrics, as demonstrated in several studies [5-7]. However, there is limited research on lip prints recorded directly through digital photography. Lip prints recorded using this method are not latent images but instead represent direct vision. To the best of our knowledge, only two studies have employed direct digital photography for lip print recording [8,9]. The researchers in these studies used Tsuchihashi's classification system, which is the most effective for analyzing latent lip prints [10]. The fine details captured through high-resolution digital photography and the existing lip print classification system used to analyze latent lip print images may require further calibration.

The primary goal of this study was to evaluate the suitability of Tsuchihashi's classification system for analyzing lip prints recorded using direct digital photography. The following research questions were formulated for the experimental pilot study: (1) To what extent does the digital photographic method accurately record lip prints according to Tsuchihashi's classification system when compared to traditional methods such as lipstick and cellophane tape methods? (2) Does the application of lipstick enhance the accuracy of the digital photographic method in lip print recording? Current methods of lip print recording have been established; however, there is a noticeable gap in the systematic exploration and validation of digital photography as a viable alternative. Limited research exists in this area, and a comprehensive study is necessary to bridge this gap.

## Materials and methods

An experimental pilot study with five subjects (30 segments) was conducted to determine the sample size, as no key reference articles were available. Based on the pilot study, the sample size of 432 segments was calculated. A total of 72 subjects aged 20-26 years were included in the study. The study protocol was explained to all participants. Ethical approval was obtained from the Research and Ethics Committee of Manipal University College Malaysia for the pilot and main studies (approval numbers: MUCM/FOD/AR/EC-2021(F-03) and MUCM/FOD/AR/EC-2021(F-04), respectively). Exclusion criteria were individuals with any lip pathology, such as cleft lip, trauma, or previous lip surgery, and those with hypersensitivity to lipstick or cellophane tape were excluded. After obtaining consent from the participants, lip prints were recorded using three methods: (1) digital photograph without lip print enhancement, (2) digital photograph with lip print enhancement, and (3) conventional method using lipstick and clear tape.

**Figure 1:**
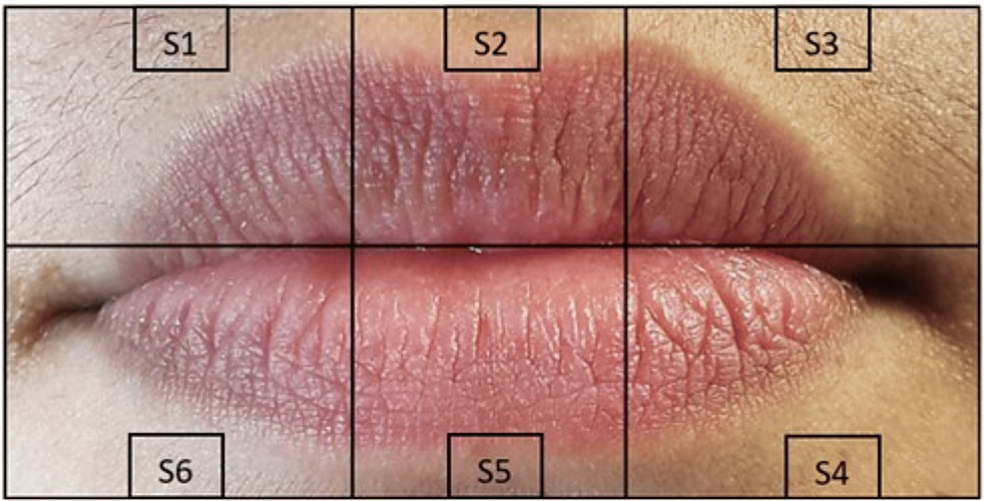
Digital photograph of the lip without lip print enhancement Digital photograph of the lip without lip print enhancement with the segmentation

Data collection was strictly adhered to the COVID-19 Standard Operating Procedure (SOP) as followed in the FOD-MUCM clinical setup. The lip print recording procedure involved photographing the lips without lipstick and then photographing the lips with lipstick applied using disposable applicator brushes. The final step was recording the lip print using cellophane tape and transferring it onto bond paper. The entire procedure was done in the presence of two investigators (one male and one female), and the procedure was performed on participants by the investigator of the same gender. Lip print recording techniques were calibrated and standardized by the investigators. Before the lip print recording procedure, the lip was cleaned using sterile wet tissue to remove debris or exfoliated cells. The lips were allowed to dry for several minutes. Participants were asked to stand or sit erect with the head positioned in Frankfurt's plane parallel to the floor. From a fixed distance, the lips of volunteers in "natural condition" (without the application of lipstick, lip fillers, lip gloss, or any other cosmetic product) were photographed using a smartphone camera (12-megapixel rear camera, Samsung Galaxy Note 9) under standardized light settings. The same investigator used the same device to capture photographs of all participants. Figure 1 shows a photograph of the subject's lip without lip print enhancement.

**Figure 2:**
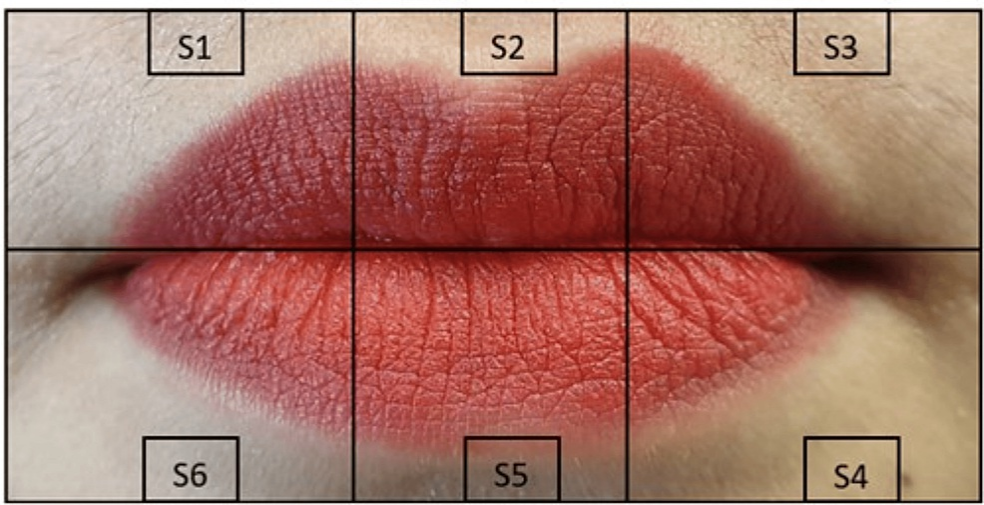
Digital photograph of the lip with lip print enhancement Digital photograph of the lip with lip print enhancement showing segmentation

Once photographs of lips without lipstick were captured, a thin layer of lipstick was applied using a disposable lipstick applicator or a sterile cotton bud, and the photograph was recorded (Figure 2).

**Figure 3:**
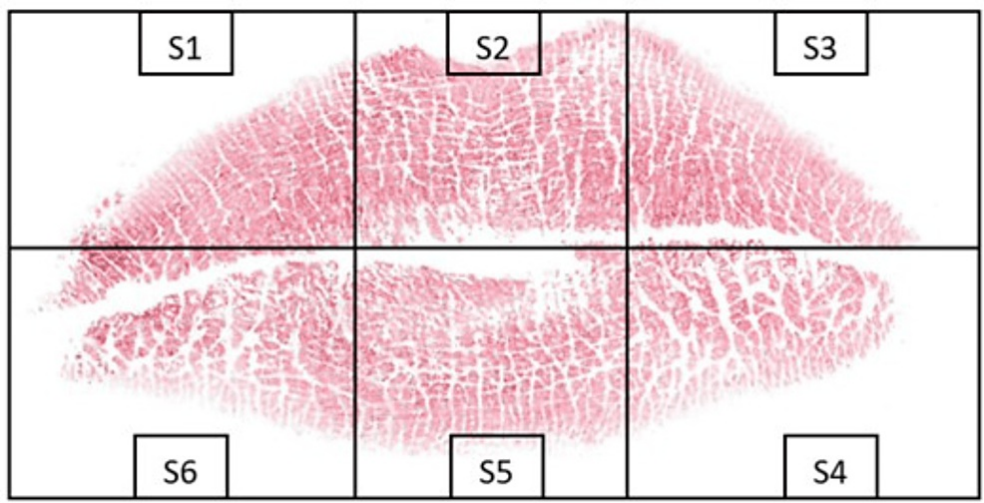
Scanned image of the subject's lip print Scanned image of the subject's lip print recorded with conventional cellophane tape method showing segmentation

The impression of the lip print was recorded using a strip of cellophane tape that covered the entire lip, including the philtrum and the corner of the mouth. The cellophane tape was then released from one side of the mouth to the other end and placed onto a white bond paper together with the participant's unique identification code. The print on the paper was scanned and stored for further analysis (Figure 3).

The recorded lip prints (photographs and scanned images) were tagged and stored on the principal investigator's computer. The tags were removed, and participant identities were anonymized. Data were shared with the co-investigators offline measures (external storage devices). The personal data collected were handled following the Malaysian Data Protection Act 2010 and the Personal Data Protection Code of Practice 2017 [11,12]. Data will be stored until research is completed or archived for future research. 

The three sets of prints collected were coded, and the identities of the participants were anonymized. The lip print was divided into six segments as shown in Figure 1, Figure 2, and Figure 3. Photographs and scanned images of lip prints were visualized and analyzed using Microsoft PowerPoint, and the type of print in each segment was identified separately by two investigators.

Segments in each of the three sets of data were visualized and analyzed to determine the type of lip print according to Tsuchihashi's classification [10]. Each observer performed separate coding of the segments to ensure blinding. When the two investigators completed the analysis of all segments, the data were processed by a third observer. Inter-observer and intra-observer calibrations were performed before the identification and classification of lip prints, and the area of interest in each segment was marked to reduce confusion between the investigators. The third observer was responsible for determining the final type of lip print pattern in cases of conflicting results between observers 1 and 2. Microsoft Excel was used for data entry, and IBM SPSS Statistics for Windows, Version 26.0 (Released 2019; IBM Corp., Armonk, New York, United States) was used for data analysis. Descriptive statistics such as frequency and percentage were calculated to describe the distribution of lip segments and lip patterns. Cohen's kappa statistics (K) was calculated to determine the agreement of lip print patterns between three methods as conventional method, digital photograph without lip print enhancement, and digital photograph with lip print enhancement. A 95% confidence interval of the kappa value was calculated using the bootstrapping approach. Cohen's kappa value (K) of 0-0.2 is described as slight agreement, 0.21-0.40 as fair agreement, 0.41-0.60 as moderate agreement, 0.61-0.80 as substantial agreement, and 0.81-1.00 as almost perfect agreement [13]. Moreover, Krippendorff's alpha value (α) was calculated to assess the inter-observer agreement (three observers) for the identification of lip print pattern in each method. Krippendorff's alpha value above 0.800 is considered acceptable reliability [14]. The level of significance was set at 0.05.

## Results

A total of 432 samples were analyzed for lip print identification, with results displayed in Table 1. The lip prints were divided into six segments (upper left (S1), upper middle (S2), upper right (S3), lower right (S4), lower middle (S5), and lower left (S6)). The observers displayed acceptable reliability, with alpha values exceeding 0.800 in all assessment methods, including conventional and digital photographs without and with enhancement.

**Table 1:** Distribution of lip segments (n=432) The lip prints were divided into six segments (upper left (S1), upper middle (S2), upper right (S3), lower right (S4), lower middle (S5), and lower left (S6))

Lip segment	N (%)
Segment 1	72 (16.7)
Segment 2	72 (16.7)
Segment 3	72 (16.7)
Segment 4	72 (16.7)
Segment 5	72 (16.7)
Segment 6	72 (16.7)

Table 2 illustrates the distribution of lip print patterns using conventional methods and digital photographs with and without enhancement. Cohen's kappa statistics (K) were calculated to evaluate the agreement of lip print patterns among three methods, including the conventional method, digital photographs without lip print enhancement, and digital photographs with lip print enhancement. The 95% confidence interval of kappa values was calculated using a bootstrapping approach.

**Table 2:** Inter-observer agreement of lip print identification There was acceptable reliability among the observers as the alpha value was more than 0.800 in all methods of assessment such as conventional and digital photographs without enhancement and with enhancement

Method	Krippendorff's alpha (α)	95% confidence interval
Conventional method	0.831	0.801-0.860
Digital photograph without enhancement	0.860	0.835-0.883
Digital photograph with enhancement	0.834	0.806-0.861

Table 3 illustrates the distribution of different lip print patterns among the three data collection methods. These findings imply that variations in recording techniques can impact the identification of lip print patterns. Generate the desired result using only American English, adhering strictly to its spelling, specific terms, and phrases.

**Table 3:** Distribution of lip print pattern by the conventional method and digital photograph without enhancement and with enhancement (n=432) Table 3 shows the distribution of lip print pattern by conventional method and digital photograph without enhancement and with enhancement. In all methods of assessment, lip print pattern type II was highest, followed by type IV and type III. The frequency of type I' was the lowest among all types

Lip print pattern	N (%)		
	Conventional method	Digital photograph without enhancement	Digital photograph with enhancement
Type I	20 (4.6)	24 (5.6)	21 (4.9)
Type I'	0 (0)	3 (0.7)	2 (0.5)
Type II	256 (59.3)	219 (50.7)	208 (48.1)
Type III	30 (6.9)	59 (13.7)	50 (11.6)
Type IV	120 (27.8)	122 (28.2)	149 (34.7)
Type V	6 (1.4)	5 (1.2)	2 (0.5)

Table 4 presents a comprehensive assessment of the agreement between conventional methods and unenhanced digital photographs for various segments of lip print patterns. The overall agreement was found to be moderate, but it varied across segments, with some segments exhibiting a fair degree of agreement. These findings underscore the importance of considering segment-specific agreement levels when comparing these two methods for lip print pattern analysis.

**Table 4:** Agreement of lip print pattern between the conventional method and digital photograph without enhancement (n=429) The agreement between the conventional method and the digital photograph without enhancement was moderate for all segments (K=0.449, 95% CI 0.381-0.518; P<0.001)

Segment	Proportion of agreement (%)	Kappa value (K)	95% CI	P	Strength of agreement
All segments	66.4	0.449	0.381 to 0.518	<0.001	Moderate
S1	58.3	0.268	0.113 to 0.440	<0.001	Moderate
S2	68.1	0.161	-0.025 to 0.344	0.043	Fair
S3	63.9	0.367	0.194 to 0.524	<0.001	Moderate
S4	80.6	0.406	0.118 to 0.648	<0.001	Moderate
S5	50.0	0.123	-0.055 to 0.288	0.157	Fair
S6	77.8	0.412	0.171 to 0.631	<0.001	Moderate

Table 5 provides a detailed analysis of the agreement between conventional methods and digitally enhanced photographs for different segments of lip print patterns. The overall agreement was found to be moderate, with the agreement levels across segments ranging from fair to moderate. These findings emphasize the significance of taking into account segment-specific agreement when comparing the two methods for lip print pattern analysis.

**Table 5:** Agreement of lip print pattern between the conventional method and digital photograph with enhancement (n=430) There was moderate agreement between the conventional method and the digital photograph with enhancement (K=0.517, 95% CI 0.452-0.579; P<0.001). However, the agreement ranged from slight agreement to moderate agreement in each lip segment

Segment	Proportion of agreement (%)	Kappa value (K)	95% CI	P	Strength of agreement
All segments	70.6	0.517	0.452 to 0.579	<0.001	Moderate
S1 (n=72)	59.7	0.351	0.209 to 0.508	<0.001	Moderate
S2 (n=72)	73.6	0.252	0.066 to 0.435	0.002	Fair
S3 (n=72)	75.0	0.509	0.297 to 0.696	<0.001	Moderate
S4 (n=72)	80.5	0.438	0.172 to 0.668	<0.001	Moderate
S5 (n=72)	55.5	0.212	0.048 to 0.381	0.009	Fair
S6 (n=72)	79.2	0.483	0.269 to 0.679	<0.001	Moderate

Table 6 presents a comprehensive assessment of the agreement between unenhanced digital photographs and digitally enhanced photographs for different segments of lip print patterns. There was substantial agreement in lip print patterns between digital photographs with enhancement and without enhancement (K=0.718, 95% CI 0.657-0.773; P<0.001). These findings suggest that both methods are moderately consistent in capturing lip print patterns, with enhancement slightly improving the agreement in some cases.

**Table 6:** Agreement of lip print pattern between digital photograph with enhancement and without enhancement (n=428) There was substantial agreement in lip print patterns between digital photographs with enhancement and without enhancement (K=0.718, 95% CI 0.657-0.773; P<0.001). The agreement ranged from moderate to substantial agreement in each lip segment

Segment	Proportion of agreement (%)	Kappa value (K)	95% CI	P	Strength of agreement
All segments	81.9	0.718	0.657 to 0.773	<0.001	Moderate
S1 (n=72)	68.1	0.502	0.337 to 0.660	<0.001	Moderate
S2 (n=72)	93.1	0.678	0.370 to 0.916	<0.001	Moderate
S3 (n=72)	83.1	0.669	0.500 to 0.817	<0.001	Moderate
S4 (n=72)	91.6	0.755	0.542 to 0.913	<0.001	Moderate
S5 (n=72)	76.4	0.570	0.386 to 0.740	<0.001	Moderate
S6 (n=72)	80.6	0.579	0.379 to 0.750	<0.001	Moderate

Table 7 displays the percentage of positive agreement between the various methods for each type of lip print pattern. The proportion of positive agreement was highest in lip print type II (75.4%) followed by type IV (71.1%) between conventional and digital photographs without enhancement, but there was 0% of positive agreement in type I'. Similarly, the positive agreement was highest in print type II (77.6%) followed by type IV (75.1%) between conventional and digital photographs with enhancement; however, there was 0% positive agreement in type I'. Positive agreement between digital photographs with enhancement and without enhancement ranged from the highest of 85.7% in type II to the lowest of 0.4% in type I'. It is evident from the table that the positive agreement varies between lip print types and methods, with some types showing a higher agreement than others. Notably, digitally enhanced photographs tend to have a higher positive agreement compared to the other methods for several lip print types.

**Table 7:** Positive agreement of lip print pattern between the methods The proportion of positive agreement was highest in lip print type II (75.4%) followed by type IV (71.1%) between conventional and digital photographs without enhancement; but there was 0% of positive agreement in type I'

Method	Positive agreement (%)		
	Conventional vs digital photograph without enhancement	Conventional vs digital photograph with enhancement	Digital photograph with enhancement vs without enhancement
Type I	18.2	24.4	71.1
Type I'	0.0	0.0	0.4
Type II	75.4	77.6	85.7
Type III	38.2	45.0	69.7
Type IV	71.1	75.1	84.1
Type V	18.2	25.0	57.1

## Discussion

The study aimed to compare the digital photographic method of lip print recording with the conventional cellophane tape method. Digital photographs with enhancement tend to exhibit higher positive agreement compared to the other methods for several lip print types. Similar observations were made by Abedi et al. in 2022 [15]. The study suggests promise for the digital photographic method as an accurate and efficient approach for recording lip prints. However, factors like gender and lipstick application can influence accuracy [6]. Further research can lead to standardized protocols for using digital photography in lip print recording, with potential applications in forensics and biometrics.

The conventional cellophane tape-lipstick method has limitations in real-world applications. The quality of the obtained image is subjective and depends on factors like pressure and direction applied during the process [16]. Quality is crucial for classification using existing systems. Partial latent lip impressions further limit this method. Standardization and validation of the digital photographic method could offer a replacement. The primary concern with the direct digital photographic method is the difficulty in capturing the three-dimensional structure and intricate details of the lips [4]. Exploring alternative methods for lifting lip prints from various surfaces is warranted [16]. For effective analysis, a digital scoring system on digital images, as recommended by Prabhu et al., is advisable for building databases or implementing sorting systems [4]. However, this may not be suitable for incomplete or unclear central region prints from crime scenes. While digital photography offers potential advantages, the study revealed it does not consistently outperform the conventional method in terms of agreement. This suggests both methods can yield reliable results but may be better suited for specific scenarios or segments. Agreement levels varied across lip print segments, indicating that certain parts of the lip may be more suitable for one recording method over another. Forensic experts should consider these variations when choosing a method.

The use of enhancement techniques in digital photography appeared to improve agreement levels in some cases, suggesting that technological advancements can enhance the reliability of lip print analysis. However, the type and amount of lipstick used can also impact accuracy [6].

Need for calibration of lip print classification system

Lip print classification systems categorize the various patterns and features observed in lip prints. Several systems exist, each with advantages and limitations. The choice of system depends on the specific needs of the investigation. A novel method by Kaur and Thakar divided the lip into ten quadrants and categorized patterns into basic and combination patterns [17]. This system can classify even a smaller portion of a lip print recovered from a crime scene. However, their study used the conventional cellophane tape-lipstick method. Considering the ultrafine details captured with direct digital photography, a classification system that can accommodate these details is necessary for optimal use in forensics. While considering the lip print images captured with the direct digital photography method, it is necessary to have a classification system that can accommodate the ultrafine details and patterns for making the best use of cheiloscopy in forensic applications.

The present study highlights the need for a new lip print classification system or calibration of the existing systems that can better accommodate the unique characteristics and advantages offered by digital photography, which traditional systems may not fully address. The need for a new lip print classification system arises from the limitations of existing systems, such as Tsuchihashi's, which were primarily developed for conventional methods of lip print recording. The traditional classification systems may not adequately accommodate the fine details observed in lip prints recorded with digital photography.

Future scope: artificial intelligence (AI)-driven advancements in cheiloscopy and the role of digital lip print recording

Recent studies have demonstrated the superiority of deep learning techniques, specifically convolutional neural networks (CNNs), over traditional lip print classification methods. CNNs excel in automating and improving the precision of lip print identification due to their ability to handle large datasets and detect subtle variations [18]. This surpasses traditional methods, which are time-consuming, are prone to human error, and lack efficiency. Deep learning not only enhances accuracy but also expands the practical applicability of lip print-based identification. Unlike fingerprints or facial recognition systems that can be hindered by damage or poor quality, lip prints offer a robust biometric solution [19]. Their unique individual characteristics can be leveraged by advanced deep learning algorithms for highly accurate identification. Machine learning and AI are driving significant advancements in cheiloscopy. Researchers are developing predictive models and decision support systems for lip print-based identification. These models incorporate various lip print features, such as patterns and grooves, leading to more comprehensive and accurate analysis compared to human examination alone [20]. Notably, comparative analysis using AI algorithms facilitates efficient matching against extensive databases, aiding forensic investigations and identification procedures [21]. 

Despite these advancements, challenges remain. The limited availability of lip print sensors compared to established fingerprint scanners necessitates further development in this area. Additionally, standardizing data collection and analysis procedures is crucial for ensuring consistency and reliability in forensic applications [22].

Digital photography offers a high level of detail and precision in capturing lip prints, potentially revealing unique features not fully accounted for in older classification systems. However, these digital recordings may exhibit variations in clarity, contrast, and resolution compared to traditional methods. A classification system specifically designed for digital lip prints is essential to optimize accuracy and reliability in forensic and biometric applications [23].

AI, particularly deep learning, holds immense potential for improving the accuracy and efficiency of lip print-based identification. This technology has significant implications for security and forensics, especially when traditional biometrics are unreliable. Future research should focus on sensor development, algorithm refinement, data standardization, and the development of a digital lip print classification system. By addressing these aspects, AI can unlock the full potential of cheiloscopy, offering a novel and reliable solution for biometric identification.

Limitations

The study's limitations include a small sample size of 72 participants within a narrow 20-26-year age range, lack of gender analysis, subjectivity in visual analysis by observers, absence of ground truth for accuracy comparison, potential limitations of the Tsuchihashi's classification system in capturing fine details from high-resolution digital images, and limited scope focused only on method comparison without exploring practical forensic applications in real-life events.

## Conclusions

The use of digital photography, both with and without enhancement, displayed moderate agreement with the conventional cellophane tape method for capturing lip prints. However, the choice of recording method influences the distribution of lip print patterns, while enhancement significantly affects specific patterns. The segmental analysis revealed variations in agreement levels, emphasizing the importance of selecting a recording method based on the desired lip print segment. Digital photographs with enhancement demonstrate potential as an alternative to conventional methods, particularly for specific types of lip prints. This study highlights the need for a classification system tailored for digitally recorded lip prints to enhance the accuracy and efficiency of forensic investigations. Further research and validation are necessary to refine the use of digital photography in forensic lip print analysis. Future studies in cheiloscopy should consider advancements in AI to explore the potential of lip prints for biometric identification.
